# New insights into epigenetic regulation of resistance to PD-1/PD-L1 blockade cancer immunotherapy: mechanisms and therapeutic opportunities

**DOI:** 10.1186/s40164-022-00356-0

**Published:** 2022-11-16

**Authors:** Mengyuan Dai, Miao Liu, Hua Yang, Can Küçük, Hua You

**Affiliations:** 1 Laboratory for Excellence in Systems Biomedicine of Pediatric Oncology, Department of Hematology and Oncology, Pediatric Research Institute, Chongqing Key Laboratory of Pediatrics, Ministry of Education Key Laboratory of Child Development and Disorders, International Science and Technology Cooperation base of Child development and Critical Disorders, National Clinical Research Center for Child Health and Disorders, Children’s Hospital of Chongqing Medical University, 136 Zhongshan Second Rd., Yuzhong District, 401122 Chongqing, China; 2 Department of Pathology, Brigham and Women’s Hospital, Harvard Medical School, Boston, USA; 3 Department of Basic Medicine and Biomedical Engineering, School of Medical, Foshan University, Foshan, China; 4 İzmir Biomedicine and Genome Center, İzmir International Biomedicine and Genome Institute, Department of Medical Biology, Dokuz Eylül University, İzmir, Türkiye; 5 İzmir International Biomedicine and Genome Institute, Dokuz Eylül University, İzmir, Türkiye; 6 Basic and Translational Research Program, İzmir Biomedicine and Genome Center, İzmir, Türkiye; 7 Department of Medical Biology, Faculty of Medicine, Dokuz Eylül University, İzmir, Türkiye

**Keywords:** PD-1/PD-L1, Immune checkpoints, Therapy resistance, Epigenetic regulation, DNA methyltransferase, Histone deacetylase, Non-coding RNA

## Abstract

Programmed cell death protein 1(PD-1) is a type of immune-inhibitory checkpoint protein, which delivers inhibitory signals to cytotoxic T cells by binding to the programmed death ligand-1 (PD-L1) displayed on the surface of cancer cells. Antibodies blocking PD-1/PD-L1 interaction have been extensively used in treatment of human malignancies and have achieved promising outcomes in recent years. However, gradual development of resistance to PD-1/PD-L1 blockade has decreased the effectiveness of this immunotherapy in cancer patients. The underlying epigenetic mechanisms need to be elucidated for application of novel strategies overcoming this immunotherapy resistance. Epigenetic aberrations contribute to cancerogenesis by promoting different hallmarks of cancer. Moreover, these alterations may lead to therapy resistance, thereby leading to poor prognosis. Recently, the epigenetic regulatory drugs have been shown to decrease the resistance to PD-1/PD-L1 inhibitors in certain cancer patients. Inhibitors of the non-coding RNAs, DNA methyltransferases, and histone deacetylases combined with PD-1/PD-L1 inhibitors have shown considerable therapeutic efficacy against carcinomas as well as blood cancers. Importantly, DNA methylation-mediated epigenetic silencing can inhibit antigen processing and presentation, which promotes cancerogenesis and aggravates resistance to PD-1/PD-L1 blockade immunotherapy. These observations altogether suggest that the combination of the epigenetic regulatory drugs with PD-1/PD-L1 inhibitors may present potential solution to the resistance caused by monotherapy of PD-1/PD-L1 immunotherapy.

## Introduction

Over the past few years, cancer immunotherapy has been at the forefront of research. A breakthrough in the treatment of advanced stage cancers has been the targeting of immunological checkpoints, particularly the interplay between programmed death 1 (PD-1) and programmed death ligand 1 (PD-L1). PD-1 inhibits T cell activation in the tumor microenvironment by binding to two ligands: PD-L1 and PD-L2 expressed on the surface of tumor cells [1–4]. PD-1/PD-L1 inhibitors can boost T cell effector activity against tumor cells by interfering with their interactions. Multiple solid tumors, including non-small cell lung cancer (NSCLC) [5], melanoma [6], triple negative breast cancer (TNBC] [7], liver cancer [8], and cervical cancer [9], as well as blood cancers like Hodgkin’s lymphoma (HL) [10] and NK/T-cell lymphoma, have been treated with PD-1/PD-L1 inhibitory drugs. However, drug resistance has gradually developed as a result of the widespread use of PD-1/PD-L1 inhibitors in cancer treatments. A majority of patients developed resistance to PD-1/PD-L1 inhibitors even if they initially showed good response to PD-1/PD-L1 therapy [11].

Among NSCLC patients treated with PD-1 inhibitors, almost half of the patients developed drug resistance [12]. The development of NSCLC was shown to be associated with genomic instability promoted by alterations in DNA methylation patterns [13, 14]. Among melanoma patients with resistance to PD-1 antibodies, the therapeutic outcomes were reported to be associated with the expression levels of certain non-coding RNAs [15]. In NSCLC and liver cancer, for instance, it was demonstrated that p53 up-regulates PD-L1 via miR-34, which could serve as a predictive biomarker for PD-1 inhibitor immunotherapy [16, 17]. Histone deacetylase 6 (HDAC6), which has the potential to be a predictive biomarker for melanoma and ovarian cancer[18, 19], also regulates epigenetic resistance to PD-1 immunotherapy in melanoma patients. Numerous studies on the epigenetic modulation of the PD-1/PD-L1 immune checkpoints have shown that a variety of epigenetic inheritance mechanisms play a significant role in the interaction between epigenetic and immune modulation [20, 21]. Here, we summarized the potential clinical benefits of epigenetic regulation on reversing resistance to PD-1/PD-L1 blockade for patients with cancers.

## Epigenetic therapies in cancer

The term ‘epigenetics’ defines all meiotically and mitotically heritable changes in gene expression that are not coded in the DNA sequence itself. Three epigenetic mechanisms, that is, DNA methylations, histone modifications, and non-coding RNAs, were shown to initiate and sustain epigenetic silencing [22]. Recently, numerous studies have suggested that epigenetic alterations play critical roles in a wide range of cancer types [23, 24]. Cancer-associated epigenetic alterations in tumors reveal potentially reversible targets for existing drugs, and for an increasing repertoire of new drugs [25, 26].

### The types of epigenetic aberrations in cancer

Methylation of the C5 position of cytosine bases in the context of CpG dinucleotides in DNA has long been recognized as an epigenetic silencing mechanism of fundamental importance [27]. The methylation of CpG sites within the human genome is generated or maintained by a number of DNA methyltransferases (DNMTs) that have multifaceted roles, including silencing of transposable elements, defense against viral sequences, and transcriptional repression of certain genes [28]. Aberrant methylation of CpG islands is a hallmark of human cancers and is observed early during carcinogenesis [29]. The aberrant silencing of genes, including tumor-suppressor genes, are linked to focal increases in methylation in promoter-associated CpG islands [30].

Post-translational histone modifications have also been defined as epigenetic processes playing critical roles in cancer development [31]. In general, hyperacetylation of histones activates transcription of genes, whereas hypoacetylation of them inactivates transcription [32]. Histone deacetylases (HDACs) regulate biological processes, which include autophagy, DNA damage repair, metabolism, apoptosis, senescence, and cell cycle control [33]. This transcriptional modulation regulates the expression of tumor suppressor, antigen-processing and presentation machinery, and tumor antigen genes that were silenced during tumorigenesis in cancer cells [34].

The role of non-coding RNAs in post-transcriptional silencing has attracted much interest. However, long noncoding RNAs (lncRNAs), in particular antisense transcripts, can also lead to mitotically heritable transcriptional silencing by the formation of heterochromatin [35, 36]. Therefore, lncRNAs might be a key trigger to direct histone modifications and DNA methylations to specific genomic loci, thereby leading to heritable and stable silencing of genes [37]. The therapeutic activation of abnormally silenced genes thus requires drugs that can target the multifaceted changes [38]. Besides, except for lncRNAs and miRNAs, circular RNAs (circRNAs) have also been identified to regulate the PD-1/PD-L1 pathway and thus participate in immune response and immunotherapy.

### Epigenetic drugs used for cancer treatment

Epigenetic alterations have fundamental roles in cancer progression characterized by reversibility and susceptibility to external factors. They are emerging as promising targets for cancer therapies. Typically, transcriptional repression is linked to the actions of DNMTs and HDACs [39]. Thus, drugs targeting these proteins can augment expression of involved genes, with many consequences for pathways downstream of this gene activation. When incorporated into DNA, DNMT inhibitors (DNMTi) act as cytidine analogues that not only cause the degradation of the target protein but also block the catalytic actions of DNMTs for triggering DNA demethylation [40, 41]. Cytosine methylation at CpG dinucleotides in DNA varies significantly in almost all cancers [42].Tumor suppressor genes were reactivated by DNMTis, which induced expression of genes that were silenced by promoter DNA methylation [43].Multiple types of tumor cells were temporarily exposed to low doses of DNMTis, which resulted in apoptosis, cell cycle inhibition, and diminished stem cell functions [44]. Due to promising clinical efficacy of DNMTis, such as 5-azacytidine and 5-aza-2-deoxycytidine (decitabine) for treating hematologic neoplasms, these DNMTi drugs were approved by FDA for treatment of myelodysplastic syndrome, a benign neoplasm, precursor of leukemia and acute myeloid leukemia [45].

For the treatment of cutaneous T-cell lymphoma (CTCL) and peripheral T-cell lymphoma, HDAC inhibitors (HDACi) have been approved [46, 47]. HDACis have pleiotropic effects, often dose and compound dependent. Some of them affect the acetylation status of non-histone and/or non-nuclear proteins or cause off-target effects like DNA damage, while others clearly cause epigenetic alterations and affect histone acetylation [48, 49]. Given the importance of epigenetic regulation in different cancer types, it is not surprising that the epigenetic targeting is becoming an attractive treatment strategy in cancer therapy. Epigenetic treatment may therefore benefit cancer patients as monotherapy or in combination with other types of traditional therapies [50].

### Resistance to PD-1/PD-L1 immunotherapy is epigenetically regulated

PD-1 is expressed not only in different kinds of tumor-infiltrating lymphocytes but also in some cancer tissues [51]. Disruption of PD-1/PD-L1 interaction inhibits immune-surveillance against cancer cells through elimination of specific or non-specific immune responses that eliminate tumor cells [52]. Globally, dozens of PD-1 or PD-L1 inhibitors were approved for marketing as they yielded good clinical responses [53–56]. However, a number of recent studies have shown that anti-PD-1/PD-L1 resistance is linked to poor drug responses in some cancer patients [57, 58]. These findings underscore the need for new approaches to combating anti-PD-1/PD-L1 resistance as well as a deeper comprehension of the underlying mechanisms. The total response rate of anti-PD-1/PD-L1 treatment for cancers is less than 20% [59].

Through inducing IFN-r activity triggered by tumor infiltrating lymphocytes (TILs), cancer cells alter PD-L1 expression [60]. DNA methylation and enhancer of zeste homolog 2 (EZH2) activity were found to induce PD-1 resistance in melanoma by inhibiting IFN-γ transcription and the RAS and PI3K pathways’ subsequent functions [61, 62]. The IFN- response was triggered by hypermetylation of PD-L1-regulating genes, limiting the activities of PD-1/PD-L1 blockade. On the other hand, cytotoxic T cell depletion is largely caused by DNA methylation. These methylation changes do have specific mechanisms which are still unclear [63].

Epigenetic processes other than DNA methylation were also reported to be associated with the resistance to PD-1 inhibitors. Modification by N6-methyladenosine (m6A) of RNA is a kind of reversible regulation. The m6A was found in about 1/3 of mRNA of eukaryotic cells, with at least 3–6 modifications in each mRNA [64]. For the melanoma cells, the fat mass and obesity-associated protein (FTO) is the first m6A demethylase identified [65]. YT521-B homology (YTH) domain family (YTHDF) proteins tend to accelerate the metabolism of mRNAs modified by m6A. The increasing FTO level decreases m6A modification of PD-1 and degradation of RNA induced by YTHDF so that the tumorigenic cells grow faster. Knocking-out FTO in the melanoma cells through in vitro experiments sensitized to IFN-γ, and increased the immune response of PD-1 inhibitor in mice melanomas. These data showed that the regulation of m6A is a potential way to eliminate the resistance to anti-PD-1 therapy [].

### The influence of epigenetic regulatory drugs on PD-1/PD-L1 immunotherapy resistance

**Fig. 1:**
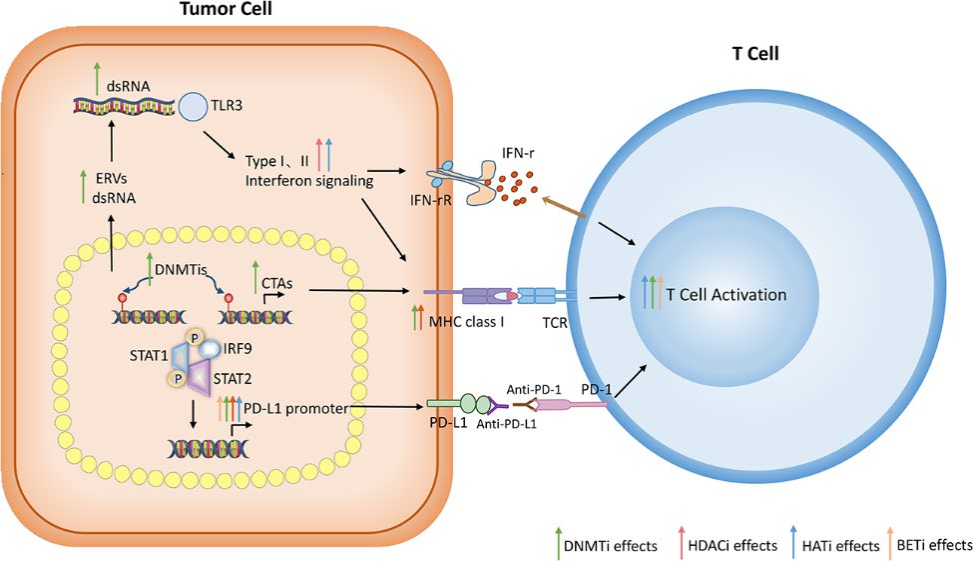
Epigenetic regulatory drugs promote anti-tumor immune signaling to synergize with PD1/PD-L1 immunotherapy

Given that there is an association between epigenetic regulation and cancer development, drugs targeting epigenetic alterations are widely used in clinical trials in combination with PD-1/PD-L1 inhibitors. The following is a list of the most frequently used epigenetic regulatory drugs: BET inhibitors, histone methyltransferase inhibitors, DNA methyltransferase inhibitors, IDH2 or TET2 inhibitors, and histone acetyltransferase inhibitors. Figure1 demonstrates the effects of epigenetic regulatory drugs on PD1/PD-L1 immunotherapy.

#### DNA methyltransferase inhibitors

DNA methylation in genomes of mammalian cells is usually generated through addition of methyl group of S-adenosylmethionine (SAM) to the upstream of promoter or cytosine residues within CpG (5’-C-phosphate-G-3’) islands at the 5’ end by DNA methyltransferases (DNMT) [67]. Genome-wide alterations in methylation levels are frequently observed in many malignant tumors with hypermethylations, especially in promoter-associated CpG islands and hypomethylations in rest of the genome[68–70].

DNA methyltransferase inhibitors (i.e., azacitidine and decitabine [5-aza-2’-deoxycytidine]) are used in the treatment of certain malignant blood cancers [71, 72]. Of note, decitabine was reported to increase the response rate to paclitaxel/carboplatin chemotherapy even when it is used in low-doses (7 mg/m^2^/day), which has more than 70% of disease control rate (DCR) in 55 patients with platinum-resistance. Indeed, the combination of cytokine-induced killer (CIK) cell immunotherapy with decitabine almost completely reversed resistance (DCR is 100%), indicating that the efficacy of immunotherapy in cancer treatment may be significantly enhanced by decitabine [73, 74].

The combination of decitabine and PD-1 inhibitor was also evaluated in a Phase 2 trial for classical Hodgkin lymphoma (cHL) [75]. Generally, the objective response rate (ORR) for anti-PD-1 monotherapy of cancer is no more than 30%, and the complete rate (CR) is less than 10% [76]. Although patients with cHL had a high ORR of 80–90% in response to PD-1 inhibitor, CR was still no more than 20% [77]. In a clinical trial with 120 refractory classical Hodgkin lymphoma patients, addition of low-dose decitabine (10 mg/d) to anti–PD-1 antibody camrelizumab yielded an ORR of 95%, and a CR of 71% which was twice superior compared to PD-1 inhibitor monotherapy. Even in patients with anti-PD-1 monotherapy resistance, ORR and CR were estimated to be 62% and CR 28%, respectively, with decitabine plus camrelizumab. The reasons for such improvements observed in therapeutic efficacy were interpreted as the ability of decitabine in increasing sensitivity to PD-1 inhibitors [78, 79].

### Histone deacetylase inhibitors

Post-translational acetylation of histones is highly dynamic and regulated by the activities of histone acetyltransferases (HATs) and histone deacetylases (HDACs) [80]. Currently, four HDAC inhibitors have been approved by FDA: vorinostat, romidepsin, belinostat, and panobinostat. HDAC inhibitors alter RNA polymerase II-driven transcription through acute hyperacetylation of histones and epigenetic regulators at the chromatin interface [32]. This transcriptional modulation includes the re-expression of genes that were silenced during tumorigenesis in cancer cells, including tumor antigens, antigen-processing and presentation machinery genes, and tumor suppressor genes [81]. These HDAC inhibitors were approved by FDA for treatment of different types of lymphoid malignancies: (1) vorinostat and romidepsin for cutaneous T-cell lymphomas [82]; (2) romidepsin and belinostat for peripheral T-cell lymphomas [83]; and (3) panobinostat in combination with dexamethasone for the treatment of multiple myeloma [83, 84].

The anti-PD-1 immunotherapy was considered as a potential way to improve clinical outcomes of T-cell lymphoma and multiple myeloma cases because PD-L1 was shown to be highly expressed in tumor cells of these patients [85, 86]. Clinical trials have also been conducted for these hematological malignancies in some countries [87, 88]; however, the therapeutic efficacy of monotherapy was not satisfactory []. In vitro experiments have revealed that HDACs might have an adjuvant function in modulation of sensitivity of the PD-1/PD-L1 signaling [90,91]. Hence, it is logical to use these two different types of drugs for more effective cancer treatment. In fact, clinical trials involving a combination of PD-1/PD-L1 and HDAC inhibitors were carried out in some cancer types such as Merkel cell carcinoma (MCC). In 2019, four MCC patients with no response to anti-PD-1 monotherapy received a combination of panobinostat and anti-PD-1 therapy, and the results showed that HDACi may increase the effectiveness of anti-PD-1/PD-L1 therapy [92]. Currently, multiple clinical trials including combination of these drugs are carried out in the USA for different cancer types (Table 1).

**Table 1:** Ongoing clinical trials evaluating the combination use of epigenetic drugs and PD-1/PDL1 immunotherapy

Trial number	Official title	Indication	Study Type
NCT04722952	PD−1 inhibitor combined with azacytidine and homoharringtonine, cytarabine, G-CSF for refractory or relapsed AML	Leukemia, AML	Phase 3
NCT04514081	The clinical trial of chidamide + decitabine + camrelizumabversusdecitabine + camrelizumab in anti-PD−1 antibody resistant patients with classical hodgkin lymphoma	Hodgkin lymphoma	Phase 2
NCT04510610	Camrelizumab plus decitabine in anti-PD−1 treatment-naive patients with relapsed/refractory classical hodgkin lymphoma	Hodgkin lymphoma	Phase 2 Phase 3
NCT03250962	SHR−1210 alone or in combination with decitabine in relapsed or refractory Hodgkin lymphoma	Hodgkin lymphoma	Phase 2
NCT04353479	PD-1 inhibitor and decitabine combination in elderly patients with relapse and refractory acute myeloid leukemia	Acute myeloid leukemia	Phase 2
NCT04651127	Anti-PD−1 antibody combined with histone deacetylase inhibitor in patients with advanced cervical cancer	Cervical cancer	Phase 1 Phase 2
NCT04512534	Sintilimab combined with chidamide in treating peripheral T cell lymphoma	Peripheral T-cell lymphoma	Phase 2
NCT02936752	Testing the safety and efficacy of the combination of the antibody pembrolizumab and entinostat in patients with myelodysplastic syndrome who are not responding to hypomethylating agents	Myelodysplastic syndrome	Phase 1
NCT04514081	The clinical trial of chidamide + decitabine + camrelizumab versus decitabine + camrelizumab in anti-PD−1 antibody resistant patients with classical hodgkin lymphoma.	Hodgkin lymphoma	Phase 2
NCT04038411	PD−1 antibody, chidamide, lenalidomide, and etoposide for relapsed or refractory NK/T Cell lymphoma	NK/T Cell lymphoma	Phase 4
NCT04040491	PD−1 Antibody, chidamide, lenalidomide and gemcitabine for peripheral T-cell lymphoma	Peripheral T-cell lymphoma	Phase 4
NCT03993626	A trial of CXD101 in combination with nivolumab in patients with metastatic microsatellite-stable colorectal cancer (CAROSELL)	Malignant colorectal neoplasms	Phase 1 Phase 2
NCT03765229	An exploratory study of pembrolizumab plus entinostat in non-inflamed stage III/IV melanoma	Melanoma	Phase 2
NCT01928576	Phase II anti-PD1 epigenetic therapy study in NSCLC (NA_00084192)	Non-small cell lung cancer	Phase 2
NCT04708470	Phase I/II trial of the combination of bintrafusp alfa (M7824), entinostat, and NHS-IL12 (M9241) in patients with advanced cancer	Metastatic solid tumor	Phase 1 Phase 2
NCT03250273	A clinical trial of entinostat in combination with nivolumab for patients with previously treated unresectable or metastatic cholangiocarcinoma and pancreatic adenocarcinoma	Pancreatic cancer	Phase 2
NCT03161223	Phase 1/2a study of anti-PD-L1 monoclonal antibody durvalumab in combination with pralatrexate and romidepsin, oral 5-aza and romidepsin, romidepsin alone, or oral 5-azacitidine for treatment of patients with relapsed and refractory PTCL	T-cell lymphoma	Phase 1 Phase 2

### Histone methyltransferase inhibitors

Histone methyltransferase (HMT) enzymes methylate residues on specific lysine residues of histones, which activate or repress transcription in a very residue and methyl group number-specific manner. Enhancer of zeste homologue 2 (EZH2), SET domain bifurcated 1 (SETDB1), and disruptor of telomeric silencing 1-like (DOT1L), all of which produce silencing marks, have all been utilized as therapeutic targets. As a result, similar to the effects of DNMTis and HDACis, inhibiting these HMTs opens up chromatin structure and activates gene expression. Only small-molecule EZH2 and DOT1L inhibitors have currently entered clinical development [93].

Melanoma suffers from decreased immunogenicity and loss of antigen presentation when the expression of EZH2 is increased. Addition of an EZH2 inhibitor to anti-CTLA-4 or IL-2 treatment reversed many of above immunosuppressive effects and significantly improved immune therapy in preclinical models [94]. Zhou et al. identified EZH2 as a therapeutic target for enhancing tumor cell antigen presentation and subsequently decreasing resistance to anti-PD-1 therapy in HNSCC [95].

A histone H3 lysine 79 (H3K79) methyltransferase called DOT1L is recruited by aberrant fusion proteins that are part of the mixed-lineage leukemia (MLL) HMT. This creates a permissive chromatin state that makes it easier for HATs and BRD4 to promote leukemia through driving transcription in an abnormal way. Pharmacological inhibition or genetic depletion of DOT1L can alleviate H3K79 methylation at the promoters of pro-inflammatory cytokines such as IL-6 and IFN-β, which is reported to be mediated by DOT1L [96]. Emilie et al. found that the combination of histone deacetylase inhibitor (SAHA) with DOT1L inhibitors (EPZ5676 or SGC0946) or BET bromodomain inhibitor (PFI-1) were efficient to partially reverse TGF-β1 effects by decreasing PD-L1 expression, suggesting that combination of epigenetic compounds might enhance clinical responses to PD-L1 [97].

### BET inhibitors

Functionally associated with transcriptional co-activators, bromodomain and extra-terminal (BET) proteins positively regulate RNA Pol II-dependent transcription at active enhancer and promoter regions. In a wide range of genetically diverse solid and hematological cancers, BET inhibitors have demonstrated broad efficacy. Suppressing Pol II-driven oncogenic transcription, particularly MYC and MYC-dependent transcriptional programs in hematological malignancies, is where BET inhibitors exert their anti-tumor effects [98].

In genetically diverse tumor models, a number of researchers have demonstrated that promoter- and enhancer-bound BET proteins are necessary for the transcription of immune checkpoint ligands PD-L1 and PD-L2. In addition, ectopic expression of PD-L1 in lymphoma was sufficient to reduce the efficacy of a BET inhibitor (i.e., JQ1) in vivo. Importantly, IRF1-driven PD-L1 expression induced by IFN-γ was also suppressed by BET inhibition, which is known to be an adaptive immune evasion mechanism. Changes in MYC expression, which may regulate PD-L1 in particular cellular contexts, had no effect on BET inhibition’s ability to suppress PD-L1 [99].

BET inhibitors have been shown to improve the efficacy of cancer immunotherapies in recent preclinical studies. In the context of MYC-driven B cell lymphoma, BET inhibitors exhibited improved activity when treated with anti-PD-1 and agonistic anti-CD137 (4-1BB) [100]. JQ1 increased the effectiveness of anti-PD-1 therapy in KRAS-driven NSCLC, which was linked to less CD4 ^+^ FOXP3 ^+^ regulatory T (Treg) cell infiltration [101]. These studies, taken together, suggest that BET bromodomain inhibitors activate the immune system of the host, which could be used to boost immune responses against tumors.

### Non-coding RNAs

Recently, non-coding RNAs have been observed to play important roles in tumor immunity [102, 103]. Non-coding RNAs are a special class of functional RNAs including microRNAs (miRNA) and long non-coding RNAs (lncRNA), which play critical roles in initiation and development of tumors [104]. miRNAs secreted by tumor-associated macrophages (TAM) results in immunosuppression and angiogenesis during cancer development [105, 106]. miR-21, miR-145, miR-100, and miR-195 were all reported to promote immunity through toll-like receptor (TLR) signaling [107–110]. Some miRNAs such as miR-21 activate the CSF1-ETS2 (V-ets erythroblastosis virus E26 oncogene homolog 2) pathway, which increases immune escape due to the elevation of the PD-L1 levels [111].

Cancer cells have multiple immune escape mechanisms to evade T-cell responses, with PD-1 pathway being a classical example. Bioinformatics analyses showed that several key lncRNAs contribute to immune escape. An important lncRNA, EPIC1, was shown to enhance methylation of the 27th amino acid in histone H3 (H3K27) by binding the enhancer of zeste homolog 2 (EZH2) protein, leading to low transcript level of IFN-γ and inhibition of IFN-JAK-STAT1 signal pathway. Cancer cells with EPIC1 overexpression have a strong resistance to PD-1 blocking treatment; and knock-out of EPIC1 can reverse this resistance [112–114]. According to the tight relationship between non-coding RNAs and resistance to PD-1 blockade, miR/PD-1 and lncRNA/PD-1 signaling pathways have been investigated as targets for potential cancer immunotherapy drugs. The compounds downregulating the miRNAs and lncRNAs targeting PD-1/PD-L1 axis may be candidates for new inhibitors [115, 116].

**Fig. 2:**
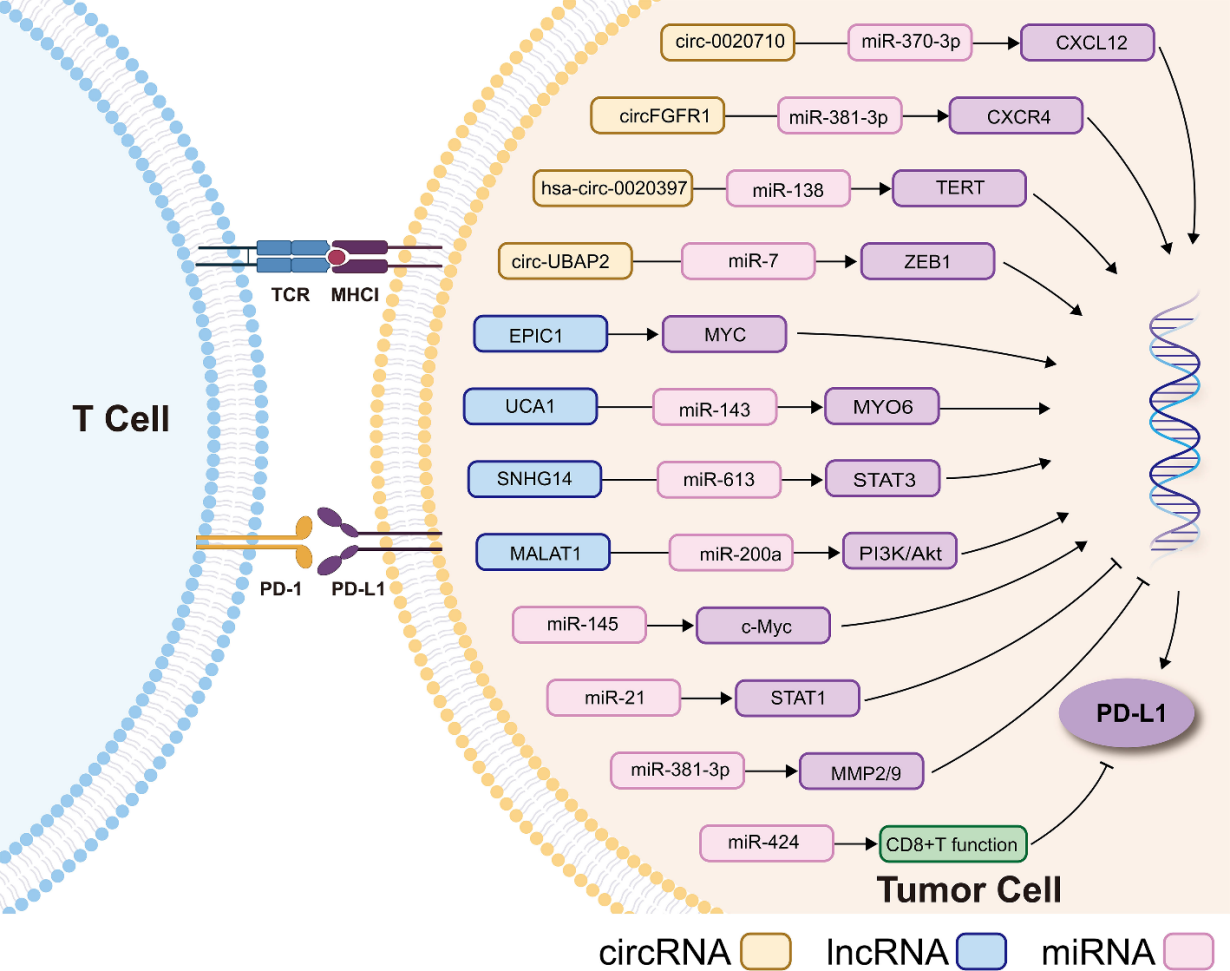
The regulation of PD-1/PD-L1 axis via non-coding RNAs

Circular RNAs (circRNAs) was firstly found as single-stranded covalently closed circular RNA molecules structures in viroids [117]. The major functions of circRNAs include regulation of gene transcription, protein binding, serving as templates for protein translation, or miRNA sponge through which binding sites for miRNAs are supplied [118]. The expression of circRNAs was observed in different diseases and dysregulated expression may affect tumor occurrence and development [119, 120]. It has been shown that circRNAs play an important role in cancer immunology by hindering the interaction of miRNA and their downstream mRNAs [121]. For example, in melanoma cells circ-0020710 binds more competitively to miR-370-3p than chemokine (C-X-C motif) ligand 12 (CXCL12) as miRNA sponge. Accumulation of CXCL12 can then recruit more immune suppressor cells which results in cytotoxic lymphocyte (CTL) depletion, and introduce the formation of immunosuppressive microenvironment which contributes to the resistance of PD-1/PD-L1 pathway blockade. Therefore, inhibition of circ-0020710 or CXCL12 increases efficacy of cancer treatment when combined with PD-1 inhibitors [122]. Furthermore, the expression of PD-1 in T cells and PD-L1 in cancer cells are found to be affected by multiple circRNAs, which also work on the development of resistance to PD-1/PD-L1 blockade. For PD-1 expression level in the T cells, circRNAs mostly act through miRNA sponging and influence downstream reaction of related mRNAs [123–125]. On the other hand, except for miRNA sponge function, alteration of cicrRNAs would affect the function of PD-L1-related transcription factors and ultimately leads to the change of PD-L1 expression in cancer cells [126–131]. Figure2 summarizes the relationship between non-coding RNAs and PD-1/PD-L1 axis in cancer cells.

## Future directions and conclusions

PD-1/PD-L1 immunotherapy has proven to be an exciting and productive area of research for treatment of malignancies; however, this type of therapy only shows long-term efficacy in a minority of patients. The technologies and drugs related to epigenetic regulation have great potential for improving the clinical efficacy of PD-1/PD-L1 immunotherapy. DNMTi, HDACi, and inhibitors of non-coding RNAs altogether are promising therapeutic agents to reverse resistance to PD1/PD-L1 blockade in a wide variety of cancers. We eagerly await results of ongoing clinical trials summarized in Table 1, which will provide information on the safety, efficacy, and potential biomarkers for these combinatory treatments. Utilizing epigenetic therapies to eliminate PD-1/PD-L1 inhibitor resistance may prove to be a safe and effective way for treating multiple types of cancer. Although combination with epigenetic drugs and inhibitors of PD-1/PD-L1 showed promising results on cancer treatment, the mechanism for such increased efficacy remains elusive, and further clinical studies are needed for elucidation of the optimal treatment conditions for the patients. The resistance induced by PD-L1 expression was regulated by two different mechanisms in cancer cells: primary resistance (intrinsic resistance) and acquired resistance. In current studies, epigenetic regulatory drugs including DNA methyltransferase inhibitors, histone deacetylase inhibitors, histone methyltransferase inhibitors, BET inhibitors, and non-coding RNAs can affect the oncogenic signaling in the cancer cells, and the resistance induced by those belongs to the primary resistance [73, 82, 97, 99]. On the other hand, some histone methyltransferase drugs, BET inhibitors and non-coding RNAs can also trigger TIL changes regulated by IFN-γ that is responsible for PD-L1 expression on immune system cells and cancer cells which reflects the mechanisms of acquired resistance [99, 112].

## Data Availability

Not applicable.

## Abbreviations

PD-1: Programmed cell death protein 1
PD-L1: Programmed death ligand-1
NSCLC: Non-small cell lung cancer
TNBC: Triple negative breast cancer
HL: Hodgkin’s lymphoma
HDAC6: Histone deacetylase 6
DNMTs: DNA methyltransferases
HDACs: Histone deacetylases
lncRNAs: Long noncoding RNAs
DNMTi: DNA methyltransferases inhibitors
CTCL: Cutaneous T-cell lymphoma
TILs: Tumor infiltrating lymphocytes
EZH2: Enhancer of zeste homolog 2
SAM: S-adenosylmethionine
CIK: Cytokine-induced killer
DCR: Disease control rate
ORR: Objective response rate
CR: complete rate
HATs: Histone acetyltransferases
MCC: Merkel cell carcinoma
HMT: Histone methyltransferase
SETDB1: SET domain bifurcated 1
MLL: Mixed-lineage leukemia
BET: Bromodomain and extra-terminal
miRNA: microRNAs
TLR: Toll-like receptor
TAM: Tumor-associated macrophage

## References

[1] Fife BT, Bluestone JA (2008). Control of peripheral T-cell tolerance and autoimmunity via the CTLA-4 and PD-1 pathways. Immunol Rev.

[2] Brahmer JR (2013). Harnessing the immune system for the treatment of non-small-cell lung cancer. J Clin Oncol.

[3] Miller JF, Sadelain M (2015). The journey from discoveries in fundamental immunology to cancer immunotherapy. Cancer Cell.

[4] Sharpe AH, Pauken KE (2018). The diverse functions of the PD1 inhibitory pathway. Nat Rev Immunol.

[5] Forde PM, Chaft JE, Smith KN, Anagnostou V, Cottrell TR, Hellmann MD, Engl (2018). Neoadjuvant PD-1 Blockade in Resectable Lung Cancer.N. J Med.

[6] Menzies AM, Johnson DB, Ramanujam S, Atkinson VG, Wong ANM, Park JJ (2017). Anti-PD-1 therapy in patients with advanced melanoma and preexisting autoimmune disorders or major toxicity with ipilimumab. Ann Oncol.

[7] Nanda R, Chow LQ, Dees EC, Berger R, Gupta S, Geva R, et al. Pembrolizumab in Patients With Advanced Triple-Negative Breast Cancer: Phase Ib KEYNOTE-012 StudyJ Clin Oncol. 2016, 34(21):2460–2467. 10.1200/JCO.2015.64.8931 PMC6816000 27138582

[8] Yau T, Hsu C, Kim TY, Choo SP, Kang YK, Hou MM (2019). Nivolumab in advanced hepatocellular carcinoma: Sorafenib-experienced Asian cohort analysis. J Hepatol.

[9] Rischin D, Gil-Martin M, González-Martin A, Braña I, Hou JY, Cho D, Falchook GS, et al. PD-1 blockade in recurrent or metastatic cervical cancer: Data from cemiplimab phase I expansion cohorts and characterization of PD-L1 expression in cervical cancer.Gynecol Oncol. 2020, 159(2): 322–328. 10.1016/j.ygyno.2020.08.026 32917410

[10] Ansell SM, Lesokhin AM, Borrello I, Halwani A, Scott EC, Gutierrez M (2015). PD-1 blockade with nivolumab in relapsed or refractory Hodgkin’s lymphoma. N Engl J Med.

[11] SynTeng.Mok NLMWLTSKA, Soo R (2017). De-novo and acquired resistance to immune checkpoint targeting. Lancet Oncol.

[12] Walsh RJ, Soo RA (2020). Resistance to immune checkpoint inhibitors in non-small cell lung cancer: biomarkers and therapeutic strategies. Ther Adv Med Oncol.

[13] Bouras E, Karakioulaki M, Bougioukas KI, Aivaliotis M, Tzimagiorgis G, Chourdakis M (2004). Gene-promoter hypermethylation as a biomarker in lung cancer. Nat Rev Cancer.

[14] Cai L, Bai H, Duan J, Wang Z, Gao S, Wang D (2019). Epigenetic alterations are associated with tumor mutation burden in non-small cell lung cancer. J immunotherapy cancer.

[15] Veronica Huber V, Vallacchi V, Fleming X, Cova HA, Dugo M (2018). Tumor-derived microRNAs induce myeloid suppressor cells and predict immunotherapy resistance in melanoma. J Clin Invest.

[16] Cortez MA, Ivan C, Valdecanas D, Wang X, Peltier HJ, Ye Y (2015). PDL1 Regulation by p53 via miR-34. J Natl Cancer Inst.

[17] Ding L, Lu S, Li Y (2020). Regulation of PD-1/PD-L1 Pathway in Cancer by Noncoding RNAs. Pathol Oncol Res.

[18] Zhicheng Hu, Rong Y, Li S, Qu S (2020). Shaobin Huang. Upregulated Histone Deacetylase 6 Associates with Malignant Progression of Melanoma and Predicts the Prognosis of Patients. Cancer Manage Res.

[19] Bitler BG, Wu S, Park PH, Hai Y, Aird KM, Wang Y (2017). ARID1A-mutated ovarian cancers depend on HDAC6 activity. Nat Cell Biol.

[20] Yan Y, Gao R, Trinh TLP, Grant MB (2017). Immunodeficiency in Pancreatic Adenocarcinoma with Diabetes Revealed by Comparative Genomics. Clin Cancer Res.

[21] Jinyang Li, Salina Yuan, Robert J Norgard, Fangxue Yan, Yu H Sun, Il-Kyu Kim, et al. Epigenetic and Transcriptional Control of the Epidermal Growth Factor Receptor Regulates the Tumor Immune Microenvironment in Pancreatic Cancer. Cancer Discov. 2021, 11(3):736-753. 10.1158/2159-8290.CD-20-0519 PMC7933070 33158848

[22] Allis C, Jenuwein T, Reinberg D, Caparros M. Epigenetics. Cold Spring Harbor Lab. Press, NY, USA, 2015.

[23] Heninger E, Krueger TE, Lang JM (2015). Augmenting antitumor immune responses with epigenetic modifying agents. Front Immunol.

[24] Ali MA, Matboli M, Tarek M, Reda M, Kamal KM, Nouh M, et al. Epigenetic regulation of immune checkpoints: another target for cancer immunotherapy? Immunotherapy. 2017, 9(1): 99–108. 10.2217/imt-2016-0111 28000527

[25] Jones PA, Ohtani H, Chakravarthy A, De Carvalho DD (2019). Epigenetic therapy in immune-oncology. Nat Rev Cancer.

[26] Qiao LY, Shen S, Liu M, Xia C, Kay JC, Zhang QL (2016). Inflammation and activity augment brain-derived neurotrophic factor peripheral release. Neuroscience.

[27] Zhou Z, Li HQ, Liu F. DNA Methyltransferase Inhibitors and their Therapeutic Potential. Curr Top Med Chem. 2018, 18(28): 2448–2457. 10.2174/1568026619666181120150122 30465505

[28] Pan Y, Liu G, Zhou F, Su B, Li Y. DNA methylation profiles in cancer diagnosis and therapeutics. Clin Exp Med. 2018, 18(1): 1–14. 10.1007/s10238-017-0467-0 28752221

[29] Takai D, Jones PA. Comprehensive analysis of CpG islands in human chromosomes 21 and 22. Proc. Natl Acad. Sci. USA 2002, 99(6): 3740–3745. 10.1073/pnas.052410099 PMC122594 11891299

[30] Jones PA, Baylin SB (2002). The fundamental role of epigenetic events in cancer. Nat Rev Genet.

[31] Lachner M, Jenuwein T (2002). The many faces of histone lysine methylation. Curr Opin Cell Biol.

[32] McClure JJ, Li X, Chou CJ (2018). Advances and Challenges of HDAC Inhibitors in Cancer Therapeutics. Adv Cancer Res.

[33] Yoon S, Eom GHHDAC, Inhibitor HDAC (2016). From Cancer to Cardiovascular Diseases Chonnam Med J.

[34] Eckschlager T, Plch J, Stiborova M, Hrabeta J. Histone Deacetylase Inhibitors as Anticancer Drugs. Int J Mol Sci. 2017 Jul 1;18(7): 1414. 10.3390/ijms18071414 PMC5535906 28671573

[35] Conte M, De Palma R (2018). Altucci L.HDAC inhibitors as epigenetic regulators for cancer immunotherapy. Int J Biochem Cell Biol.

[36] Zhou Y, Liu M, Li J (2015). Impact of V-ets erythroblastosis virus E26 oncogene homolog 1 gene polymorphisms upon susceptibility to autoimmune diseases: A meta-analysis. Med (Baltim).

[37] Maria Angelica Cortez 1 (2015). Cristina Ivan 1, David Valdecanas 1, Xiaohong Wang 1, Heidi J Peltier 1, Yuping Ye.PDL1 Regulation by p53 via miR-34. J Natl Cancer Inst.

[38] Kensler TW, Spira A, Garber JE, Szabo E, Lee JJ, Dong Z (2016). Transforming Cancer Prevention through Precision Medicine and Immune-oncology. Cancer Prev Res (Phila).

[39] Stresemann C, Brueckner B, Musch T, Stopper H, Lyko F (2006). Functional diversity of DNA methyltransferase inhibitors in human. cancer cell lines Cancer Res.

[40] Hervouet E, Peixoto P, Delage-Mourroux R, Boyer-Guittaut M, Cartron PF (2018). Specific or not specific recruitment of DNMTs for DNA methylation, an epigenetic dilemma. Clin Epigenetics.

[41] Angelique Bruyer, Ken Maes, Laurie Herviou, Alboukadel Kassambara , Anja Seckinger, Guillaume Cartron, et al.DNMTi/HDACi combined epigenetic targeted treatment induces reprogramming of myeloma cells in the direction of normal plasma cellsBr J Cancer. 2018, 118(8):1062-1073. 10.1038/s41416-018-0025-x PMC5931098 29500406

[42] Clements EG, Mohammad HP, Leadem BR, Easwaran H, Cai Y, Van Neste L (2012). DNMT1 modulates gene expression without its catalytic activity partially through its interactions with histone-modifying enzymes. Nucleic Acids Res.

[43] Baylin SB, Jones PA. Epigenetic Determinants of Cancer. In: Allis CD, Carparros M-L, Jenuwein T, Reinberg D, editors. Epigenetics, 2nd edition. Cold Spring Harbor, NY: Cold Spring Harbor Laboratories, 2015. 10.1101/cshperspect.a019505 PMC5008069 27194046

[44] Tsai HC, Li H, Van Neste L, Cai Y, Robert C, Rassool FV (2012). Transient low doses of DNA-demethylating agents exert durable antitumor effects on hematological and epithelial tumor cells. Cancer Cell.

[45] Matei D, Fang F, Shen C, Schilder J, Arnold A, Zeng Y (2012). Epigenetic resensitization to platinum in ovarian cancer. Cancer Res.

[46] Duvic M, Talpur R, Ni X, Zhang C, Hazarika P, Kelly C (2007). Phase 2 trial of oral vorinostat (suberoylanilide hydroxamic acid, SAHA) for refractory cutaneous T-cell lymphoma (CTCL). Blood.

[47] VanderMolen KM, McCulloch W, Pearce CJ, Oberlies NH (2011). Romidepsin: a natural productrecently approved for cutaneous T-cell lymphoma. J Antibiot.

[48] Falkenberg KJ, Johnstone RW (2014). Histone deacetylases and their inhibitors in cancer, neurological diseases and immune disorders. Nat Rev Drug Discov.

[49] Kaminskas E, Farrell A, Abraham S, Baird A, Hsieh LS, Lee SL (2005). Approval summary: azacitidine for treatment of myelodysplastic syndrome subtypes. Clin Cancer Res.

[50] Cameron EE, Bachman KE, Myohanen S, Herman JG, Baylin SB (1999). Synergy of demethylation and histone deacetylase inhibition in the re-expression of genes silenced in cancer. Nat Genet.

[51] Salmaninejad A, Valilou SF, Shabgah AG, Aslani S, Alimardani M (2019). PD-1/PD-L1 pathway: Basic biology and role in cancer immunotherapy. J Cell Physiol.

[52] Abiko K, Matsumura N, Hamanishi J, Horikawa N, Murakami R, Yamaguchi K (2015). IFN-γ from lymphocytes induces PD‐L1 expression and promotes progression of ovarian cancer. Br J Cancer.

[53] Okazaki T, Honjo T. PD-1 and PD-1 ligands: from discovery to clinical application. Int Immunol. 2007 Jul;19(7):813–24. 10.1093/intimm/dxm057 17606980

[54] Constantinidou A, Alifieris C, Trafalis DT. Targeting Programmed Cell Death – 1 (PD-1) and Ligand (PD-L1): A new era in cancer active immunotherapy. Pharmacol Ther. 2019, 194: 84–106. 10.1016/j.pharmthera.2018.09.008 30268773

[55] Hayashi H, Nakagawa K (2020). Combination therapy with PD-1 or PD-L1 inhibitors for cancer.Int. J Clin Oncol.

[56] Armand, P., Shipp, M. A., Ribrag, V., Michot, J.-M., Zinzani, P, et al. 2016. Programmed death-1 blockade with pembrolizumab in patients with classical Hodgkin lymphoma after brentuximab vedotin failure. Journal of Clinical Oncology, 34(31): 3733-3739. 10.1200/JCO.2016.67.3467 PMC5791838 27354476

[57] Armand P, Shipp MA, Ribrag V, Michot J-M, Zinzani P (2016). Programmed death-1 blockade with pembrolizumab in patients with classical Hodgkin lymphoma after brentuximab vedotin failure. J Clin Oncol.

[58] Asano T, Meguri Y, Yoshioka T, Kishi Y, Iwamoto M, Nakamura M, et al. PD-1 modulates regulatory T-cell homeostasis during low-dose interleukin-2 therapy. Blood. 129(15): 2186–2197. 10.1182/blood-2016-09-741629 PMC5391624 28151427

[59] Wakabayashi G, Lee YC, Luh F, Kuo CN, Chang WC, Yen Y. Development and clinical applications of cancer immunotherapy against PD-1 signaling pathway. J Biomed Sci. 2019, 26(1): 96. 10.1186/s12929-019-0588-8 PMC6894306 31801525

[60] Yuan Y, Adam A, Zhao C, Chen H. Recent Advancements in the Mechanisms Underlying Resistance toPD-1/PD-L1 Blockade Immunotherapy. Cancers (Basel). 2021, 13(4): 663. 10.3390/cancers13040663 PMC7915065 33562324

[61] Kim KH, Roberts CW. Targeting EZH2 in cancer. Nat Med. 2016, 22(2): 128–134. 10.1038/nm.4036 PMC4918227 26845405

[62] Wu X, Gu Z, Chen Y, Chen B, Chen W, Weng L (2019). Application of PD-1 Blockade in Cancer Immunotherapy. Comput Struct Biotechnol J.

[63] Emran AA, Chatterjee A, Rodger EJ (2019). Targeting DNA methylation and EZH2 activity to overcome melanoma resistance to immunotherapy J. Trends Immunol.

[64] Stephanie Oerum, Vincent Meynier, Marjorie Catala, Carine Tisné. A comprehensive review of m6A/m6Am RNA methyltransferase structures. Nucleic Acids Res. 2021, 49(13):7239-7255. 10.1093/nar/gkab378 PMC8287941 34023900

[65] Yang S, Wei J, Cui YH, Park G, Shah P, Deng Y (2019). m(6)A mRNA demethylase FTO regulates melanoma tumorigenicity and response to anti-PD-1 blockade. Nat Commun.

[66] Seungwon Yang, Jiangbo Wei, Yan-Hong Cui, Gayoung Park, Palak Shah , Yu Deng. m6A mRNA demethylase FTO regulates melanoma tumorigenicity and response to anti-PD-1 blockadeNat Commun. 2019, 10(1):2782. 10.1038/s41467-019-10669-0 PMC6592937 31239444

[67] Moore LD, Le T, Fan G. DNA methylation and its basic function. Neuropsychopharmacology. 2013, 38(1): 23–38. 10.1038/npp.2012.112 PMC3521964 22781841

[68] Jin B, Robertson KD (2013). DNA methyltransferases, DNA damage repair, and cancer J. Adv Exp Med Biol.

[69] Wong KY, Chim CS (2015). DNA methylation of tumor suppressor protein-coding and non-coding genes in multiple myeloma J. Epigenomics.

[70] Can K, Hu X, Jiang B, Klinkebiel D, Geng H, Gong Q (2015). Global promoter methylation analysis reveals novel candidate tumor suppressor genes in natural killer cell lymphoma. Clin Cancer Res.

[71] Kaminskas E, Farrell AT, Wang YC (2005). FDA drug approval summary: azacitidine(5-azacytidine, VidazaTM)for injectable suspension[J]. Oncologist.

[72] Gore SD, Jones C, Kirkpatrick P (2006). Decitabine[J]. Nat Rev Drug Discov.

[73] Zhang Y, Mei Q, Liu Y, Li X, Brock MV, Chen M (2017). The safety efficacy, and treatment outcomes of a combination of low-dose decitabine treatment in patients with recurrent ovarian cancer. Oncoimmunology.

[74] Stresemann C, Lyko F (2008). Modes of action of the DNA methyltransferase inhibitors azacytidine and decitabine. Int J Cancer.

[75] Jing Nie, Chunmeng Wang, Yang Liu, Qingming Yang, Qian Mei, Liang Dong, et al. Addition of Low-Dose Decitabine to Anti-PD-1 Antibody Camrelizumab in Relapsed/Refractory Classical Hodgkin Lymphoma. J Clin Oncol. 2019, 37(17):1479-1489. 10.1200/JCO.18.02151 31039052

[76] Wu X, Gu Z, Chen Y, Chen B, Chen W, Weng L (2019). Application of PD-1 Blockade in Cancer Immunotherapy. Comput Struct Biotechnol J.

[77] Zhang Y, Hao L, Zhao Z, Yang X, Wang L, Liu S (2020). .Immuno-DNA binding directed template-free DNA extension and enzyme catalysis for sensitive electrochemical DNA methyltransferase activity assay and inhibitor screening. Analyst.

[78] Nie J, Wang C, Liu Y, Yang Q, Mei Q, Dong L (2019). Addition of Low-Dose Decitabine to Anti–PD-1 Antibody Camrelizumab in Relapsed/Refractory Classical Hodgkin Lymphoma. J Clin Oncol.

[79] Gourd E. New treatment for relapsed or refractory Hodgkin’s lymphoma. Lancet Oncol. 2019, 20(6): e298. 10.1016/S1470-2045(19)30289-X 31085049

[80] Bates SE, Eisch R, Ling A (2015). Romidepsin in peripheral and cutaneousT-cell lymphoma: mechanistic implications from clinical and correlative data. Br J Haematol.

[81] Eckschlager T, Plch J, Stiborova M, Hrabeta J. Histone Deacetylase Inhibitors as Anticancer Drugs. Int J Mol Sci. 2017, 18(7): 1414. 10.3390/ijms18071414 PMC5535906 28671573

[82] Lopez AT, Bates S, Geskin L (2018). Current Status of HDAC Inhibitors in Cutaneous T-cell Lymphoma. Am J Clin Dermatol.

[83] Mulvey E, Ruan J. Biomarker-driven management strategies for peripheral T cell lymphoma. J Hematol Oncol. 2020, 13(1): 59. 10.1186/s13045-020-00889-z PMC7245625 32448357

[84] Bantscheff M, Hopf C, Savitski MM, Dittmann A, Grandi P (2011). Chemoproteomics profiling of HDAC inhibitors reveals selective targeting of HDAC complexes. Nat Biotechnol.

[85] Lee B-H, Park Y, Kim JH, Kang K-W, Lee SJ, Kim SJ, et al. PD-L1 expression in bone marrow plasma cells as a biomarker to predict multiple myeloma prognosis: developing a nomogram-based prognostic model. Sci Rep. 2020 Jul 28;10(1):12641. 10.1038/s41598-020-69616-5 PMC7387472 32724129

[86] Tiemann M, Samoilova V, Atiakshin D, Buchwalow I. Immunophenotyping of the PD-L1-positive cells in angioimmunoblastic T cell lymphoma and Hodgkin disease. BMC Res Notes. 2020 Mar 7;13(1):139. 10.1186/s13104-020-04975-w PMC7060537 32143684

[87] Jelinek T, Paiva B, Hajek R (2018). Update on PD-1/PD-L1 Inhibitors in Multiple Myeloma.Front Immunol. Nov.

[88] Tomassetti S, Chen R, Dandapani S.The role of pembrolizumab in relapsed/refractory primary mediastinal large B-cell lymphoma.Ther Adv Hematol. 2019, 22;10: 2040620719841591. 10.1177/2040620719841591 PMC6477766 31040936

[89] Oliva S, Troia R, D’Agostino M, Boccadoro M, Gay F (2018). Promises and Pitfalls in the Use of PD-1/PD-L1 Inhibitors in Multiple Myeloma. Front Immunol.

[90] Xinyi Tu B, Qin Y, Zhang C, Zhang M, Kahila S, Nowsheen, et al. PD-L1 (B7-H1) Competes with the RNA Exosome to Regulate the DNA Damage Response and Can Be Targeted to Sensitize to Radiation or Chemotherapy. Mol Cell. 2019 Jun 20;74(6):1215–1226. 10.1016/j.molcel.2019.04.005 PMC6737939 31053471

[91] Yang Gao NT, Nihira X, Bu C, Chu J, Zhang A, Kolodziejczyk (2020). Acetylation-dependent regulation of PD-L1 nuclear translocation dictates the efficacy of anti-PD-1 immunotherapy. Nat Cell Biol.

[92] Selma Ugurel I, Spassova J, Wohlfarth C, Drusio A, Cherouny A, Melior (2019). MHC class-I downregulation in PD-1/PD-L1inhibitor refractory Merkel cell carcinoma and its potential reversal byhistone deacetylase inhibition: a case seriesCancer. Immunol Immunother.

[93] Yang C, Zhang J, Ma Y, Wu C, Cui W, Wang L (2020). Histone methyltransferase and drug resistance in cancers. J Exp Clin Cancer Res.

[94] Zingg D, Arenas-Ramirez N, Sahin D, Rosalia RA, Antunes AT, Haeusel J (2017). The Histone Methyltransferase Ezh2 Controls Mechanisms of Adaptive Resistance to Tumor Immunotherapy. Cell Rep.

[95] Liye Zhou T, Mudianto X, Ma R, Riley R, Uppaluri (2020). Targeting EZH2 Enhances Antigen Presentation, Antitumor Immunity, and Circumvents Anti-PD-1 Resistance in Head and Neck Cancer. Clin Cancer Res.

[96] Spencer AMaiques-Diaz,GJ, Lynch JT, Ciceri F, Williams EL, Fabio MR, Amaral (2018). Enhancer Activation by Pharmacologic Displacement of LSD1 from GFI1 Induces Differentiation in Acute Myeloid Leukemia. Cell Rep.

[97] Emilie Evanno J, Godet N, Piccirilli J, Guilhot S, Milin JM, Gomber, et al. Tri-methylation of H3K79 is decreased in TGF-β1-induced epithelial-to-mesenchymal transition in lung cancer. Clin Epigenetics. 2017, 9: 80. 10.1186/s13148-017-0380-0 PMC5549304 28804523

[98] Filippakopoulos P, Knapp S (2014). Targeting bromodomains: epigenetic readers of lysine acetylation. Nat Rev Drug Discov.

[99] Abruzzese MP (2016). Inhibition of bromodomain and extra-terminal (BET) proteins increases NKG2D ligand MICA expression and sensitivity to NK cell-mediated cytotoxicity in multiple myeloma cells: role of cMYC-IRF4-miR-125b interplay. J Hematol Oncol.

[100] Abruzzese MP, Bilotta MT, Fionda C, Zingoni A, Soriani A, Vulpis E, et al. BET bromodomain inhibition cooperates with PD-1 blockade to facilitate antitumor response in kras-mutant non-small cell lung cancer. Cancer Immunol. Res.2018, 6, 1234–1245. 10.1158/2326-6066.CIR-18-0077 PMC6170698 30087114

[101] Dennis O, Adeegbe S, Liu, Maureen M, Hattersley M, Bowden CW, Zhou S, Li (2018). BET bromodomain inhibition cooperates with PD-1 blockade to facilitate antitumor response in kras-mutant non-small cell lung cancer.Cancer Immunol. Res.

[102] Zhou Y, Kong Y, Fan W, Tao T, Xiao Q, Li N, et al. Principles of RNA methylation and their implications for biology and medicine. Biomed Pharmacother. 2020 Nov;131:110731. 10.1016/j.biopha.2020.110731 32920520

[103] Bhan A, Soleimani M, Mandal SS (2017). Long Noncoding RNA and Cancer: A New Paradigm. Cancer Res.

[104] Jiang MC, Ni JJ, Cui WY, Wang BY, Zhuo W (2019). Emerging roles of lncRNA in cancer and therapeutic opportunities. Am J Cancer Res.

[105] Huber V, Vallacchi V, Fleming V (2018). Tumor-derived microRNAs induce myeloid suppressor cells and predict immunotherapy resistance in melanoma [J]. J Clin Invest.

[106] Ghafouri-Fard S, Esmaeili M, Taheri MH19, lncRNA: Roles in tumorigenesis.Biomed Pharmacother. 2020, 123: 109774. 10.1016/j.biopha.2019.109774 31855739

[107] Bica-Pop C, Cojocneanu-Petric R, Magdo L, Raduly L, Gulei D, Berindan-Neagoe I. Overview upon miR-21 in lung cancer: focus on NSCLC. Cell Mol Life Sci. 2018, 75(19): 3539–3551. 10.1007/s00018-018-2877-x PMC11105782 30030592

[108] Wang X, He Y, Mackowiak B. Gao B. MicroRNAs as regulators, biomarkers and therapeutic targets in liver diseases. Gut. 2021, 70(4): 784–795. 10.1136/gutjnl-2020-322526 33127832

[109] Ravegnini G, Cargnin S, Sammarini G, Zanotti F, Bermejo JL, Hrelia P, et al. Prognostic Role of miR-221 and miR-222 Expression in Cancer Patients: A Systematic Review and Meta-Analysis. Cancers (Basel). 2019, 11(7): 970. 10.3390/cancers11070970 PMC6678869 31336701

[110] Nallasamy P, Chava S, Verma SS, Mishra S, Gorantla S, Coulter DW (2018). PD-L1, inflammation, non-coding RNAs, and neuroblastoma: Immuno-oncology perspective. Semin Cancer Biol.

[111] Sanchez Calle A, Kawamura Y, Yamamoto Y, Takeshita F, Ochiya T. Emerging roles of long non-coding RNA in cancer. Cancer Sci. 2018, 109(7): 2093–2100. 10.1111/cas.13642 PMC6029823 29774630

[112] Milad Ashrafizadeh A, Zarrabi K, Hushmandi V, Zarrin ER, Moghadam A, Zabolian, et al. PD-1/PD-L1 axis regulation in cancer therapy: The role of long non-coding RNAs and microRNAs. Life Sci. 2020, 256: 117899. 10.1016/j.lfs.2020.117899 32504749

[113] Su M, Xiao Y, Tang J, Wu J, Ma J, Tian B, et al. Role of lncRNA and EZH2 Interaction/Regulatory Network in Lung Cancer. J Cancer. 2018, 9(22):4156–4165. 10.7150/jca.27098 PMC6277609 30519315

[114] Joshi M, Rajender S (2020). Long non-coding RNAs (lncRNAs) in spermatogenesis and male infertility. Reprod Biol Endocrinol.

[115] Palanisamy Nallasamy S, Chava SS, Verma S, Mishra S, Gorantla, Don W, Coulter (2018). PD-L1, inflammation, non-coding RNAs, and neuroblastoma: Immuno-oncology perspective. Semin Cancer Biol.

[116] Ashrafizadeh M, Zarrabi A, Hushmandi K, Zarrin V, Moghadam ER, Zabolian A (2020). PD-1/PD-L1 axis regulation in cancer therapy: The role of long non-coding RNAs and microRNAs. Life Sci.

[117] Sanger HL, Klotz G, Riesner D, et al. Viroids are single-stranded covalently closed circular RNA molecules existing as highly base-paired rod-like structures. 10.1073/pnas.73.11.3852 PMC431239 1069269

[118] Huang A, Zheng H, Wu Z (2020). Circular RNA-protein interactions: functions, mechanisms, and identification. Theranostics.

[119] Kristensen LS, Andersen MS, Stagsted LVW (2019). The biogenesis, biology and characterization of circular RNAs. Nat Rev Genet.

[120] Xu X, Zhang J, Tian Y, et al. CircRNA inhibits DNA damage repair by interacting with host gene. Mol Cancer. 2020 Aug 24;19(1):128. 10.1186/s12943-020-01246-x PMC7446195 32838810

[121] Tanaka E, Miyakawa Y, Kishikawa T (2019). Expression of circular RNA CDR1-AS in colon cancer cells increases cell surface PD-L1 protein levels. Oncol Rep.

[122] Wei CY, Zhu MX, Lu NH (2020). Circular RNA circ_0020710 drives tumor progression and immune evasion by regulating the miR-370-3p/CXCL12 axis in melanoma. Mol Cancer.

[123] Wang J, Zhao X, Wang Y (2020). circRNA-002178 act as a ceRNA to promote PDL1/PD1 expression in lung adenocarcinoma. Cell Death Dis.

[124] Wang X, Yao Y, Jin M (2020). Circ-0001068 is a novel biomarker for ovarian cancer and inducer of PD1 expression in T cells. Aging.

[125] Zhang PF, Pei X, Li KS (2019). Circular RNA circFGFR1 promotes progression and anti-PD-1 resistance by sponging miR-381-3p in non-small cell lung cancer cells. Mol Cancer.

[126] Hong W, Xue M, Jiang J (2020). Circular RNA circ-CPA4/ let-7 miRNA/PD-L1 axis regulates cell growth, stemness, drug resistance and immune evasion in non-small cell lung cancer (NSCLC). J Exp Clin Cancer Res.

[127] Li L, Zhang Q, Lian K (2020). Circular RNA circ_0000284 plays an oncogenic role in the progression of non-small cell lung cancer through the miR-377-3p-mediated PD-L1 promotion. Cancer Cell Int.

[128] Yang J, Jia Y, Wang B (2021). Circular RNA CHST15 sponges miR-155-5p and miR-194-5p to promote the immune escape of lung cancer cells mediated by PD-L1. Front Oncol.

[129] Yang Z, Chen W, Wang Y (2021). CircKRT1 drives tumor progression and immune evasion in oral squamous cell carcinoma by sponging miR-495-3p to regulate PDL1 expression. Cell Biol Int.

[130] Jiang Z, Hou Z, Liu W (2021). Circ-Keratin 6c promotes malignant progression and immune evasion of colorectal cancer through microRNA-485-3p/programmed cell death receptor ligand 1 axis. J Pharmacol Exp Ther.

[131] Xu G, Zhang P, Liang H (2021). Circular RNA hsa_circ_0003288 induces EMT and invasion by regulating hsa_circ_0003288/miR-145/PD-L1 axis in hepatocellular carcinoma. Cancer Cell Int.

