# Correction: New insights into epigenetic regulation of resistance to PD-1/PD-L1 blockade cancer immunotherapy: mechanisms and therapeutic opportunities

**DOI:** 10.1186/s40164-022-00359-x

**Published:** 2022-12-19

**Authors:** Mengyuan Dai, Miao Liu, Hua Yang, Can Küçük, Hua You

**Affiliations:** 1 Laboratory for Excellence in Systems Biomedicine of Pediatric Oncology, Department of Hematology and Oncology, Pediatric Research Institute, Chongqing Key Laboratory of Pediatrics, Ministry of Education Key Laboratory of Child Development and Disorders, International Science and Technology Cooperation Base of Child Development and Critical Disorders, National Clinical Research Center for Child Health and Disorders, Children’s Hospital of Chongqing Medical University, 136 Zhongshan Second Road, Yuzhong District, Chongqing, 401122 China; 2 Department of Pathology, Brigham and Women’s Hospital, Harvard Medical School, Boston, USA; 3 Department of Basic Medicine and Biomedical Engineering, School of Medical, Foshan University, Foshan, China; 4 İzmir International Biomedicine and Genome Institute, Dokuz Eylül University, İzmir, Türkiye; 5 Basic and Translational Research Program, İzmir Biomedicine and Genome Center, İzmir, Türkiye; 6 Department of Medical Biology, Faculty of Medicine, Dokuz Eylül University, İzmir, Türkiye

**Correction: Experimental Hematology & Oncology (2022) 11:101** https://doi.org/10.1186/s40164-022-00356-0

In this article [1] the wrong figure appeared as Fig. 1, The Fig. 1 should have appeared as shown below. 4th affiliation was inadvertently supplied and published, this has been removed. Citation for following references are missing.

**Fig. 1:**
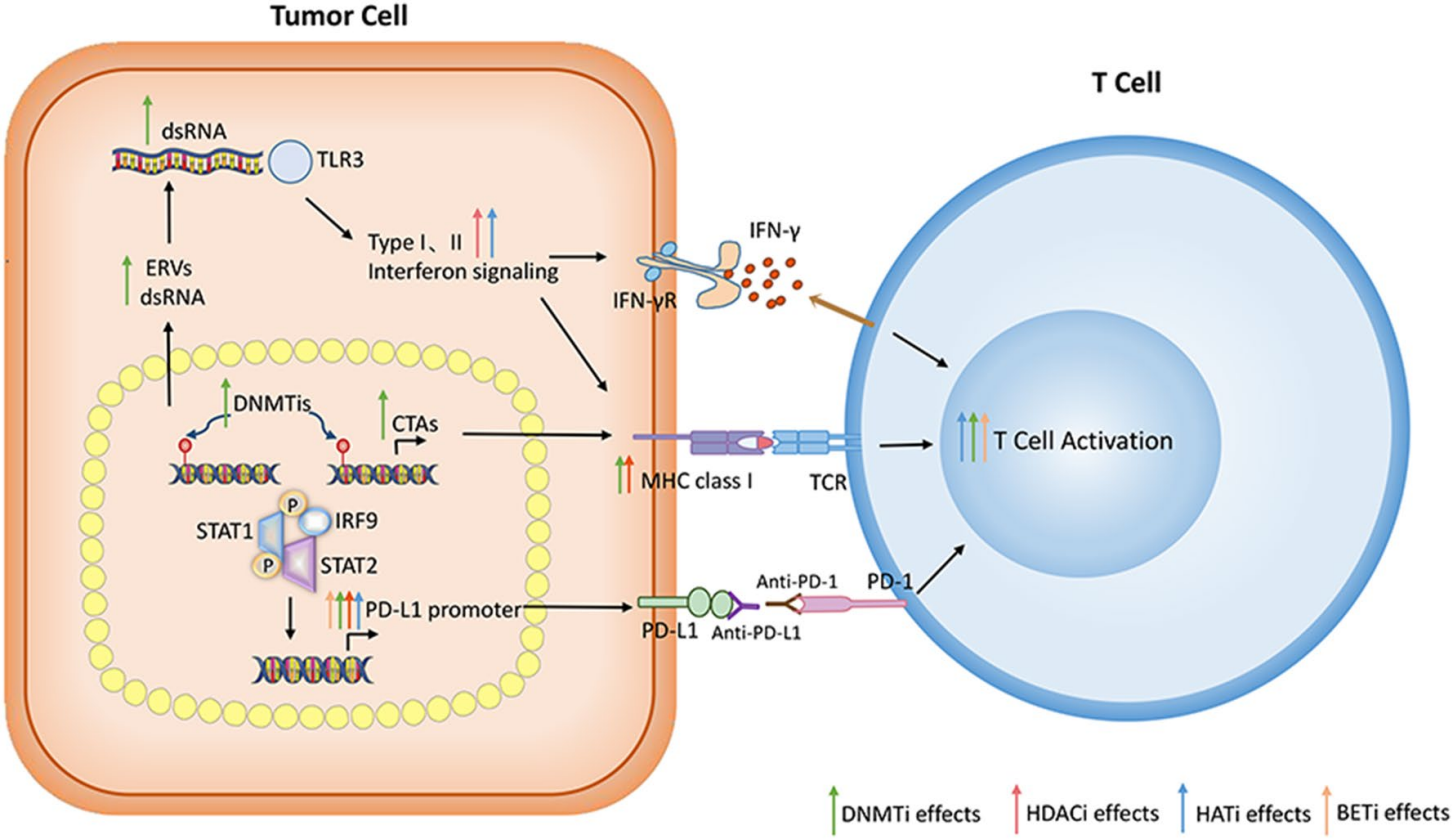
Epigenetic regulatory drugs promote anti-tumor immune signaling to synergize with PD1/PD-L1 immunotherapy

The original article has been corrected.

List of Citation and References missing in the article:

Li X, Cheng Y, Zhang M, Yan J, Li L, Fu X, et al. Zhang L. Activity of pembrolizumab in relapsed/refractory NK/T-cell lymphoma. Journal of hematology & oncology. 2018 Dec;11(1):1–8.

Merry CR, Forrest ME, Sabers JN, Beard L, Gao XH, Hatzoglou M, et al. DNMT1-associated long non-coding RNAs regulate global gene expression and DNA methylation in colon cancer. Hum Mol Genet. 2015 Nov 1;24(21):6240–53.

Das PM, Singal R. DNA methylation and cancer. J Clin Oncol. 2004 Nov 15;22(22):4632–42.

Zhang Q, Wang S, Chen J, Yu Z. Histone Deacetylases (HDACs) Guided Novel Therapies for T-cell lymphomas. Int J Med Sci. 2019 Jan 29;16(3):424–442.

Chen PL, Roh W, Reuben A, Cooper ZA, Spencer CN, Prieto PA, et al. Analysis of Immune Signatures in Longitudinal Tumor Samples Yields Insight into Biomarkers of Response and Mechanisms of Resistance to Immune Checkpoint Blockade. Cancer Discov. 2016 Aug;6(8):827–37.

Giri AK, Aittokallio T. DNMT Inhibitors Increase Methylation in the Cancer Genome. Front Pharmacol. 2019 Apr 24;10:385.

Maouche N, Kishore B, Bhatti Z, Basu S, Karim F, Sundararaman S, et al. Panobinostat in combination with bortezomib and dexamethasone in multiply relapsed and refractory myeloma; UK routine care cohort. PLoS One. 2022 Jul 7;17(7):e0270854.

Chen X, Liu X, Zhang Y, Huai W, Zhou Q, Xu S, et al. Methyltransferase Dot1l preferentially promotes innate IL-6 and IFN-β production by mediating H3K79me2/3 methylation in macrophages. Cell Mol Immunol. 2020 Jan;17(1):76–84.

Ebine K, Kumar K, Pham TN, Shields MA, Collier KA, Shang M, et al. Interplay between interferon regulatory factor 1 and BRD4 in the regulation of PD-L1 in pancreatic stellate cells. Sci Rep. 2018 Sep 5;8(1):13225.

Hogg SJ, Vervoort SJ, Deswal S, Ott CJ, Li J, Cluse LA, et al. BET-Bromodomain Inhibitors Engage the Host Immune System and Regulate Expression of the Immune Checkpoint Ligand PD-L1. Cell Rep. 2017 Feb 28;18(9):2162–2174.

Mathsyaraja H, Thies K, Taffany DA, Deighan C, Liu T, Yu L, et al. CSF1-ETS2-induced microRNA in myeloid cells promote metastatic tumor growth. Oncogene. 2015 Jul;34(28):3651–61.

Guo W, Wang Y, Yang M, Wang Z, Wang Y, Chaurasia S, et al. LincRNA-immunity landscape analysis identifies EPIC1 as a regulator of tumor immune evasion and immunotherapy resistance. Sci Adv. 2021 Feb 10;7(7):eabb3555.
